# High-dose dobutamine stress magnetic resonance perfusion imaging at 3.0 Tesla

**DOI:** 10.1186/1532-429X-15-S1-P211

**Published:** 2013-01-30

**Authors:** Rolf Gebker, Alexander Berger, Christopher Schneeweis, Thomas Hucko, Bernhard Schnackenburg, Christoph Klein, Sebastian Kelle, Eckart Fleck

**Affiliations:** 1 German Heart Institute Berlin, Berlin, Germany; 2 Philips Health Care, Hamburg, Germany

## Background

Dobutamine stress magnetic resonance (DSMR-wall motion) has been established as a valuable tool for the detection of inducible wall motion abnormalities. Additional perfusion imaging during DSMR (DSMR-perfusion) was shown to improve sensitivity for the detection of myocardial ischemia. Current experience is based on 1.5T only, mainly due to insufficient image quality of steady state free precession (SSFP) cine imaging at higher field strengths. Recently, image quality and diagnostic accuracy of SSFP sequences was significantly improved by dual-source parallel radiofrequency (RF) transmission for 3.0T. We examined whether the addition of myocardial perfusion imaging during DSMR at 3.0T is feasible and whether it provides incremental benefit for the evaluation of CAD.

## Methods

DSMR-wall motion and -perfusion were combined in 48 consecutive patients on 3.0T clinical systems (Philips Achieva and Ingenia, both equipped with a dual-source RF transmission system) applying a standard high dose dobutamine-atropine protocol. Perfusion images were acquired in three short axis views during maximum stress. Wall motion and perfusion images were interpreted sequentially by two blinded readers with invasive coronary angiography serving as the reference standard.

## Results

Assessment of wall motion and perfusion at rest and during stress were feasible in 98% of patients. One patient (2%) developed atrial fibrillation during dobutamine infusion and the examination had to be terminated. Two patients (4%) developed wall motion abnormalities before reaching target heart rate and DSMR-perfusion was performed at this time. Mean dobutamine dose was 32±8 µg/kg/min, atropine was given in 28 (58%) patients. Interobserver agreement was very good for DSMR-wall motion (κ 0.88) and -perfusion (κ 0.83). The prevalence of CAD (>70% stenosis) was 64% and involved 45 coronary territories. On a patient basis, diagnostic accuracy of DSMR-wall motion and DSMR-perfusion were similar (Table). DSMR-wall motion correctly identified 28 (62%) territories supplied by a stenotic coronary artery, whereas DSMR-perfusion correctly identified significantly more ischemic territories (n=38, 84%, P<0.002). The ability to recognize a patient as having multivessel disease by more than one territory demonstrating an ischemic reaction was improved significantly by use of DSMR-perfusion (91% vs. 36%, p=0.03, Figure).

**Table 1:** Diagnostic accuracy of DSMR-wall motion and DSMR-perfusion

	Sensitivity			Specificity			Accuracy		
Coronary stenosis ≥50%	DSMR-wall motion	DSMR-perfusion	P	DSMR-wall motion	DSMR-perfusion	P	DSMR-wall motion	DSMR-perfusion	P

All patients	27/36 (75)	30/36 (83)	0.25	10/11 (91)	10/11 (91)	1	37/47 (79)	40/47 (85)	0.38

Coronary stenosis ≥70%									

All patients	26/30 (87)	28/30 (93)	0.5	15/17 (88)	14/17 (82)	1	41/47 (87)	42/47 (89)	0.38

values are n (%), P indicates the significance level for differences between DSMR-wall motion and DSMR-perfusion

**Figure 1:**
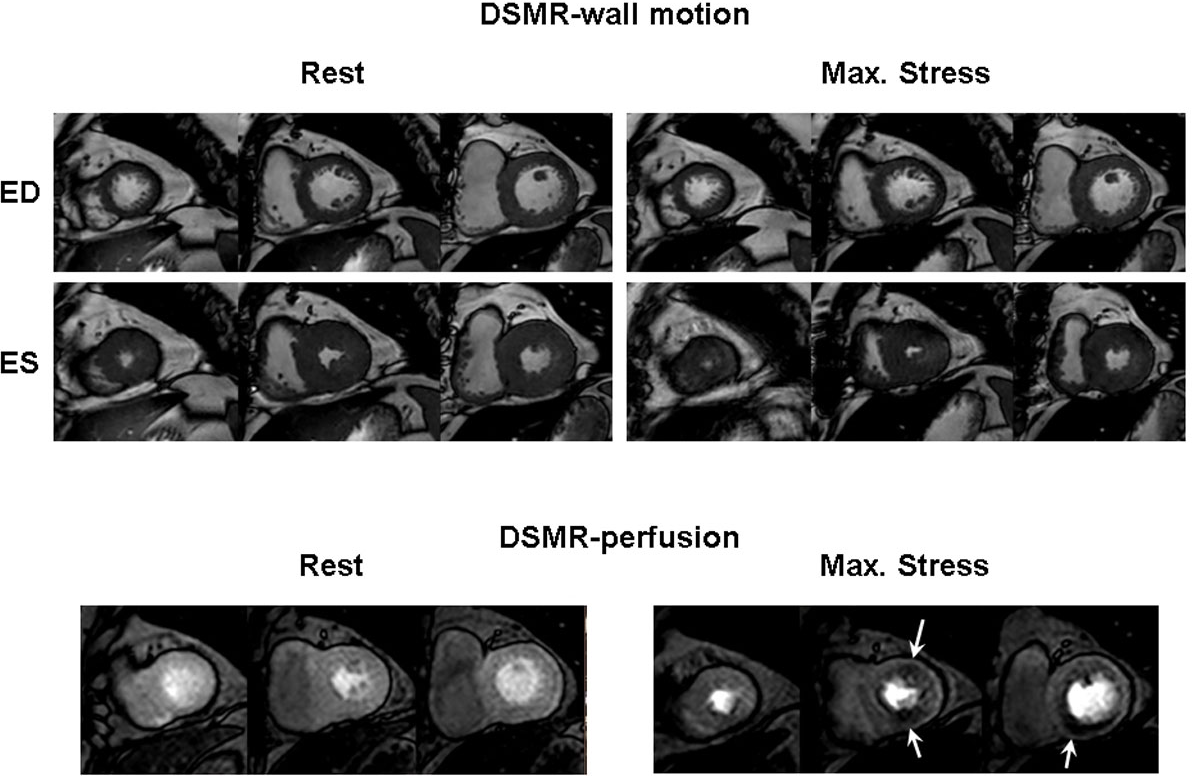
Patient with stenoses of the LAD- and RCA-territory showing false-negative DSMR-wall motion and true-positive DSMR-perfusion (white arrows), ED=end diastolic, ES=end systolic

## Conclusions

DSMR-perfusion is feasible with a high procedural success rate at 3.0T applying dual-source RF transmission and has additional diagnostic value compared to DSMR-wall motion regarding the extent of stress inducible ischemia.

## Funding

none

